# Methodological considerations in using the Network Scale Up (NSU) for the estimation of risky behaviors of particular age-gender groups: An example in the case of intentional abortion

**DOI:** 10.1371/journal.pone.0217481

**Published:** 2019-06-11

**Authors:** Maryam Zamanian, Farzaneh Zolala, Ali Akbar Haghdoost, Saeide Haji-Maghsoudi, Zeynab Heydari, Mohammad Reza Baneshi

**Affiliations:** 1 Department of Epidemiology, School of Health, Arak University of Medical Sciences, Arak, Iran; 2 Social Determinants of Health Research Center, Institute for Futures Studies in Health, Kerman University of Medical Sciences, Kerman, Iran; 3 HIV/STI Surveillance Research Center, and WHO Collaborating Center for HIV Surveillance, Institute for Futures Studies in Health, Kerman University of Medical Sciences, Kerman, Iran; 4 Department of Biostatistics, School of Public Health, Hamadan University of Medical Sciences, Hamadan, Iran; 5 Non-Communicable Diseases Research Centre, Fasa University of Medical Sciences, Fasa, Iran; 6 Modeling in Health Research Center, Institute for Futures Studies in Health, Kerman University of Medical Sciences, Kerman, Iran; Middlesex University, UNITED KINGDOM

## Abstract

**Background:**

Network Scale Up (NSU) is a promising tool for size estimation of sensitive issues. In this study we investigated the important methodological considerations to employ this method for estimating behaviors, such as abortion, which happens in a particular age-gender group.

**Methods:**

We recruited 1250 males and 1250 females aged 18 to 50. Abortion rate was calculated through direct question and NSU methodology. The NSU was applied on three sub-samples (male, female and aggregate). Integrating replies to 25 reference groups, we estimated the network size (C) of respondents and its age-gender structure. To calculate the part of network that is subject to abortion, we compared two approaches: proportional and data based. The Visibility Factor (VF) was calculated through 222 females who had abortion. Direct estimate was considered as gold standard.

**Results:**

Using C’s derived from proportional method, the Relative Bias (RB) in the male and female samples was 33% and 84%. Applying the data-based C’s, the RB in the gender-specific and aggregate samples was 5% and 2%.

**Conclusion:**

The proportional method overestimates the prevalence. The data-based method to calculate the C is superior. The determination of the age-sex distribution of the network and the specific VF is essential.

## Introduction

Abortion is an important contributing factor in female’s health that could result in irreparable effects and even death of the mother. Abortion is associated with legal restrictions as well as religious and social stigma in many societies [1–3]. In Iran, with the Islamic culture, intentional abortion is banned [4]. In addition, 2012 onwards, the policy of the country has been based on the increase in the fertility rate, which imposed further limitations in practice of abortion. Almost all (98%) unsafe abortions, the third cause of maternal death [5], occur in developing countries, moreover in contrast to other causes, all complications and deaths related to unsafe abortions could be thoroughly preventable [6]. While the policy-makers need accurate data, to reduce unsafe abortion and to improve maternal health, these barriers make it difficult to obtain valid statistics [7, 8].

The Network Scale Up (NSU) is an established tool in the size estimation of the hidden groups. This method has a practical appeal as the data are collected from the members of the general population and the participants respond on behalf of their network rather than themselves [9].

A prime in the NSU studies is the calculation of the network size. Once network size (shown by C) is estimated, a sample from the general population is recruited and asked to describe the number they know (shown by m) in specific risky population, for example number of those provide sex in exchange of money. The NSU assumes that the prevalence of a behavior in the network of a randomly selected sample (m/ C) can be generalized to the whole population (e/ t). Here, ‘m’ is the number of individuals in the hidden group known by the respondent, ‘C’ is the respondent’s network size, ‘e’ is the real size of the hidden group and ‘t’ is the size of the total population [10].

One of the assumptions of NSU method is that respondents are aware of sensitive characteristics of their acquaintances [11]. Incomplete knowledge of the respondents leads to an under-reporting of ‘m’ and the underestimation of the size [12]. Therefore, a correction factor, known as the Visibility Factor (VF), is required to adjust the crude estimates. For example, a VF of 50% indicates that crude NSU estimates should be doubled.

Over the past few years, our research team has designed several NSU projects at the local and national level. We have published more than 20 manuscripts in this field and have estimated the number of those who used illicit drugs [13] and alcohol [14], and those who participated in risky sexual behaviors [15]. In addition to that, to explore the practicality of the technique in other settings, we determined the completeness of our cancer registry [16].

In the light of our ongoing experience in the field of NSU, we were aware that a number of methodological considerations should be considered when analyzing behaviours which happen in a particular age-gender population. For example, abortion is a behavior related to females in reproductive age. In the NSU analysis, those who form the network size ‘C’ should have the potential to become a member of ‘m’. We have shown Iranian residents, in average, know 308 persons. The question is how many of these 308 ones are Females At Reproductive Age (FARA).

Another methodological challenge is about selection of respondents. It has been shown that male respondents, relative to female respondents, were 45% less likely to report or to know at least one person who had an abortion [17]. This raises the question that whether selection of respondents from one gender leads to bias in size estimation or not.

The aim of this manuscript is to address the methodological challenges in size estimation of a risky behavior which happens in a particular age-gender group. Intentional abortion is used as an example.

## Methods

### Sampling procedure

In 2016, a cross sectional study was conducted in the city of Kerman (the capital of the largest province in Iran). This city has a population of 580,000 people, of which 27% (i.e. 160,000) are Female At Reproductive Age (FARA). We recruited 1,275 male and 1,275 female respondents proportionate to the age distribution of the general population. At the first step, seeking expert views, we classified the city into three socioeconomic zones. This was followed by a random selection of five regions in each zone (15 regions in total). In each of these regions, 170 pedestrians (85 females and 85 males) were recruited.

In gender-matched interviews, aims of the study were explained and those consented verbally enrolled the study. Due to cultural issues we decided not to obtain written consent. In Iran’s setting, asking for written or signed consent lead to significant attrition rate. Male and female respondents were interviewed by trained male and female interviewers respectively. Interviewing was happened both in the morning and afternoon hours. Hereafter, these two samples are named male and female samples, respectively. In the female sample, 1020 subjects were aged 18 to 50 (subject to abortion) and the rest were more than 50 years of age. We merged these two data sets (i.e. male and female samples) to get an aggregate sample.

### Direct estimation

We assumed that those who are at risk of attempting an induced abortion are subject to abortion. We only asked the females aged 18 to 50 (n = 1020 cases) whether they had any intentional abortion within the last year. It is defined as the elective termination of pregnancy without medical justification. To secure their confidentiality, this section was self-administered and the questionnaires were put into a ballot box. The proportion of the positive replies was multiplied to the total population of the females at a reproductive age (here 160,000) to get the annual number. As we guaranteed the confidentiality, we assumed that replies to direct question is not prone to any bias and therefore this figure was used as the gold standard.

### Process of calculation and calibration of the network size (C_agg_, C_m_ and C_f_)

#### Samples used

To calculate the aggregate network size (C_agg_), we used the replies of all the 2550 subjects. To determine the network size of the males (C_m_) and females (C_f_), a stratified analysis was applied where the male and female samples were analyzed independently (n = 1275 in each). In all of the three analyses, ‘t’ was set at 580,000, and the process was started with 25 reference groups.

#### Combination of replies

The process of calculation of network size and part of it subject to abortion is summarized in Table 1. The reference group method was applied to estimate the average network size [18]. We selected multiple reference groups (13 female and 12 male first names) with known sizes (shown by e_j_). The names were selected from the civil registry list based on the following criteria: the proportion in the general population ranging from 0.1% to 4%, not changing their popularity in the recent decades, not being two-part and not being used for both genders.

**Table 1:** Description of studies implements to estimate the Annual Intentional Abortion Rate (AIAR) and visibility factor.

Network size estimation study			VF study (previously published)	
sample	n	Calculation of part of C subject to abortion	N	Relations analysed
Aggregate	2550	Starts with 25 questions “how many people you know named X” C was multiplied to 0.27	75	All relations
		Starts with 13 questions “how many female you know named X aged 18–50”		
Male	1275	Starts with 25 questions “how many people you know named X” C was multiplied to 0.27	75	Male relations
		Starts with 13 questions “how many female you know named X aged 18–50”		
female	1275	Starts with 25 questions “how many people you know named X” C was multiplied to 0.27	75	Female relations
		Starts with 13 questions “how many female you know named X aged 18–50”		

Applying the standard definition of ‘know’, participants were asked about the number of people they know with any of the selected names. We explained participants that they should count the number of people “you know them and they know you by sight and name; you have had some contact with them in the past two years; and you can contact them in future”. To estimate C, we combined replies and the information of the reference groups via Eq 1. Here, ‘m_ij_’ shows the number known by the respondent ‘i’ in the reference group ‘j’.

$$C_{i}=\left(t*\sum m_{ij}\right)/\sum \;e_{j}$$ Eq 1

#### Calibration process

Respondents might not accurately recall all the reference groups [19]. There are some evidences that suggest that respondents usually undercount the number they know in larger reference groups [20]. To exclude the ineligible reference groups, we back calculated the size of all reference groups. The ratio of the back calculated size to the real size for all the reference groups was calculated. Then, an absolute logarithm based two of all ratios were calculated. The reference group with the worst ratio was eliminated. Then, the C was re-estimated using the remaining reference groups. This process was repeated until all the absolute logarithm based two of all ratios remained less than 1 [19].

### Determination of the part of C_agg_, C_m_ and C_f_that is subject to abortion

To determine the part of network size that is subject to abortion two approaches were tried: proportional and data based.

#### Proportion based method

Here, we assumed that the distribution of the respondents' network size is the same as that of the population (named proportional approach). Based on civil registry statistics, 27% of Kermanian residents were FARA. Therefore, C_agg_, C_m_ and C_f_ values were multiplied with 0.27 (named C_agg.prop_, C_m.prop_ and C_f.prop_).

#### Data based method

In our questionnaire, in addition to asking the respondents about the number of their acquaintances in each of the 25 reference groups, we asked them to classify their reply with respect to the age group (<18, 18–50, >50). In the data-based scenario, we restricted our analyses to the replies to 13 female names in the age group 18–50 (named C_agg.data_, C_m.data_ and C_f.data_). Here, ‘t’ was set at 160,000 and then Eq 1 and the iteration methodology explained above was applied.

### Estimation of the aggregate and gender specific VF

The method frequently used to calculate the VF is known as the game of contact [21, 22]. In this method, a sample from the hidden group is selected. Through face to face interviews, they are asked a series of questions, such as “how many male/ female relatives/ friends do you have?” “How many of them are aware that you are engaged in this kind of risky behavior?” To calculate the VF, the total number of the aware acquaintances is divided by the total number of acquaintances.

We already calculated the VF for abortion using game of contact methodology [22]. For the sake of completeness, its methodology is explained briefly [17]. Approaching the private and public health centers, a total of about 75 Kermanian female who had an intentional abortion within the last year were recruited. The data were collected using a structured interview instrument administered by a trained female interviewer. We divided the entire social network into a list of comprehensive relationships. We asked them “how many people you know in each relationship category?” “How many of them are aware that you had an abortion in the last year? To secure the confidentiality, these interviews are performed in private rooms. To calculate the aggregate VF, the total number of the aware acquaintances was divided by the total number of acquaintances (VF_agg_). To estimate the gender specific VF, categories related to the male and female relations were analyzed separately (shown by VF_m_ and VF_f_), [17].

### NSU estimation

We ask all 2550 recruited subjects “how many female you know in Kerman city who had an intentional abortion in the last year. To help the participant and to enhance the accuracy of the replies, the question was asked separately for relatives, husband’s or wives’ relatives (in case the subject is married) and acquaintances (involving neighbor, friend, colleague, etc.). Summation of replies to these three categories was used as final ‘m’. The data were collected through structured face-to-face interviews. We analysed aggregated data (n = 2550) as well as gender specific data where sample size in male and female samples were 1275. Eq 2 was applied to estimate the crude size of the hidden groups and its standard errors where, ‘t’ was set at 160,000 [23]. The crude size was divided by the VF to adjust the size for visibility (Eq 3).

$$e_{\mathrm{crude}}=\left(t*\sum m_{i}\right)/\sum C_{i}\;SE(e_{crude})=Sqrt(t*\frac{e_{crude}}{\sum C_{i}})$$ Eq 2

$$e_{adj}=\{\left(t*\sum m_{i}\right)/\sum C_{i}\}*\left\{1/VF\right\}$$ Eq 3

### Comparison of the estimates

We considered the results of the direct method as the gold standard. We defined the Relative Bias (RB) as the difference between NSU estimates (by changing the parameters detailed above) and the direct estimate divided by the direct estimate: $RB=\frac{NSU\;estimate-Direct\;estimate}{Direct\;estimate}$.

The study protocol was approved by the Ethical Committee of the Kerman University of Medical Sciences (ir.kmu.rec.1394.223). The analyses were performed using the R software and Microsoft Excel (2007).

### NSU study (N = 2,550 in aggregate and 1,275 in gender specific samples)

- In all analyses, iteration was applied to exclude ineligible reference groups
- In approaches started with 25 reference groups, t was set at 580,000
- In approaches started with 13 reference groups, t was set at 160,000

### VF study (n = 75 female who had abortion in the last year)

- Relationships were categorized as male (such as uncle, brother …) and female (i.e. sister aunt…)
- A series of questions “how many people you know in each category”? “How many of them are aware”? were asked

### Direct study (n = 1,020)

- In the direct estimation method, using female sample, we only asked those subject to abortion (1020 out of 1275) whether they had intentional abortion in the past year.

## Results

### Sample characteristics

In the female sample, the mean (SD) of age was 36.46 years (13.96). About 72% of the participants were married. In addition, 43.2% of the females had an academic education (more than 12 years of education). In the male sample, the mean (SD) of age was 36.58 years (14.22). About 64% of the participants were married. In addition, 47.8% of them had an academic education.

### Direct estimate of intentional abortion

In the direct method, the prevalence of the positive reply was 0.98% (about 10 per 1000 Female At Reproductive Age (FARA)). This is corresponded to 1550 intentional abortions per year in Kerman city.

### Aggregate and gender specific network Size (C_agg_, C_m_ and C_f_)

The average network size of the Kermanian residents was estimated at C_agg_ = 177. The average network size of the females was slightly higher than the males (C_f_ = 186 and C_m_ = 169).

### Determination of the part of C_agg_, C_m_ and C_f_ that is subject to abortion

Regardless of the study population (aggregate or gender specific), the proportional method suggested a smaller figure than the data based method (Table 2). In the aggregate sample, the proportional and data based approaches suggested that 48 and 77 of the C_agg_ were females at a reproductive age, respectively.

**Table 2:** Influence of the method of estimation of the network and the study population on the size estimation of the annual number of intentional abortions.

Scenario	Method to find C	VF	Network size	Annual Number	Rate per 1000 FARA	RB
**Aggregate**	**Proportional**	V_agg_ = 0.08	C_tprop_ = 48	2550 (2436, 2666)	15.9	0.64
**Aggregate**	**Data based**	V_agg_ = 0.08	C_tdata_ = 77	1590 (1518, 1662)	9.9	0.02
**Female**	**Proportional**	V_f_ = 0.11	C_fprop_ = 50	2852 (2684, 3020)	17.8	0.84
**Female**	**Data based**	V_f_ = 0.11	C_fdata_ = 105	1440 (1356, 1524)	9	-0.07
**Male**	**Proportional**	V_m_ = 0.05	C_mprop_ = 45	2051 (1901, 2201)	12.8	0.32
**Male**	**Data based**	V_m_ = 0.05	C_mdata_ = 63	1465 (1357, 1573)	9.2	-0.05

VF: Visibility Factor

FARA: Female At Reproductive Age

RB: Relative Bias, calculated with respect to the direct estimation

Restricting our attention to the female population, the difference between the two approaches was remarkable. While, the proportional method suggests that only 50 of the C_f_ are subject to abortion, the corresponding figure in the data based approach was about two times higher, at 105. In the male population, the difference between the approaches was less profound (45 in the proportional versus 63 in the data based approaches).

### Estimation of the aggregate and gender specific VF

We have shown that the visibility of the intentional abortion was 0.08. The visibility among the female relations was more than two times higher than the male (VF_f_ = 0.11 vs. VF_m_ = 0.05), [17].

### NSU estimations

As summarized in Table 2, the proportional method consistently provides estimates which were much higher that the direct estimate (2051 in male, 2852 in female and 2550 in aggregate samples).

On the other hand, the results of the data based approach, was much closer to the direct estimate. The Confidence Intervals (CI) of the aggregate and male samples were (1518, 1662) and (1357, 1573) which covered the direct estimate (i.e. 1550). This CI corresponding to the female sample (1356, 1524) was marginally close to our direct estimate (i.e. 1550).

### Comparison of scenarios

In the proportional method, the Relative Bias (RB) was at least 32% (in the male sample) and was as high as 84% (in the female sample). The data-based method provides promising results. The RB in the male and female samples was about 5%. In the aggregate sample, the RB was as low as 2%. In the aggregate sample, the point estimate was 1590, corresponding to 9.9 abortions per 1000 FARA.

## Discussion

Recruiting 1275 male and 1275 female pedestrians, we illuminated how NSU method should be applied in the case of behaviors related to a particular age-gender group. Integrating replies to 25 reference groups, we estimated the network size of respondents and its age-gender structure. It is worth mentioning that there might be overlap between networks of respondents. However, it has been shown that overlapping does not lead to biased estimation [24].

To calculate the part of our network that is subject to abortion, we compared two approaches: proportional and data based. Our results revealed that the data based method provided a higher C than the proportional based method. The ratios of these two approaches were 1.60 in the aggregate sample (77 over 48), 2.10 in the female sample (105 over 50) and 1.40 in the male sample (63 over 45).

The NSU formula implies that higher the C, lower our estimate. The proportional based approach gives a lower C and therefore, overestimates the true size of the intentional abortion (RB: 64% in the aggregate data, 84% in the female data and 33% in the male data).That is why the analysis of the female data resulted in the lowest and the highest estimates (2852 in the proportional and 1440 in the data based methods).These results support the hypothesis that the determination of the age-sex distribution of the network size is essential.

We should mention that the assumption that ‘the age-gender distribution of the general population fits that of respondents’ seems unlikely. This assumption means that, for example, the proportion of female at reproductive age in the general population is the same as that in network size of respondents. However, this might not be the case. This is because there is some evidence to suggest that people are usually connected with their peer groups, i.e. young female are more likely to connect to other young females [25].

However, we believe that it is very likely that other NSU size estimation studies which reported age-gender specific statistics had made this assumption. Although they have not fully explained their methodology, it is highly likely that they had followed proportional method, as the composition of network size had not been provided.

Another hypothesis tested in this study was whether the analysis of the female respondents, in comparison with the male respondents, results in a higher estimate of abortion size. Surprisingly, the CIs derived in these two samples had overlapped indicating no significant difference. The females were more likely to know about the abortions in their network (0.10 versus 0.03 in average). However, this was neutralized by their higher C and visibility. In comparison with the direct method, the NSU analysis of the male and female analysis corresponded only to a 5% relative bias.

In the case of the stigmatized behaviors, the direct method is prone to underestimation. In our experience, the results of the direct and NSU methods were fairly close suggesting the internal validity of our study. In the direct method, we only asked females whether they had abortion in the last year. We did not ask men about their wives. This is because we knew that visibility of intentional abortion for husbands was 90% [17]. However, in NSU study, respondents from both genders were recruited to compare estimates of gender-specific with aggregate sample.

Closeness of results of direct and NSU study might be due to the issue that for a direct estimation, a self-administered questionnaire at the end of the NSU interview was submitted to the respondents and the forms were returned through a ballot box, hence, the anonymity was maximized. In the case of stigmatized behaviours, direct question is subject to underestimation of the true size. However, our results indicates that consideration of the methodological issues reduces prestige bias and provides useful statistics. It also implies the usefulness of the NSU. Two important superiorities of the NSU over the direct method are that the former requires a much lower sample size and allows an estimation of the size of several hidden groups in one single study. The drawback is the necessity of the estimation of the network size and correction factors.

We have previously performed national studies to calculate average network size of Iranian population, as well as prevalence of several risky behaviors at a national level. The average network size (C) of the Iranian population was estimated at 308 [18]. We approached nearly 7500 pedestrians and asked about their number of their acquaintances in 23 reference groups. Reference groups include first names, specific jobs, and some diseases with known prevalence. Ineligible reference groups were excluded in an iterative process as explained in the methods section. One of limitations of that study was that age-gender distribution of network size was not determined.

One of the aims of the national study was to estimate prevalence of abortion. At the time we analysed our national data, no study applying a concrete methodology reported the VF for abortion. Therefore, we used an ad-hoc approach to calculate the VF. We approached 34 midwives and gynecologists across the country and asked them about the minimum and maximum level of visibility of intentional abortion in Iran’s culture [26]. Intentional abortions were considered as those without any medical indication. None of these experts were involved in the main study. A questionnaire was emailed to them in which the meaning of visibility was explained as the proportion of acquaintances of a case that are aware that the case had abortion. In our national study, we used the proportional approach to determine the network size (i.e. C). That is, we multiplied the average network size of Iran (i.e. 308) by 0.27 and get a value of 83. Seeking the experts’ opinion, the minimum and maximum possible bounds for the VF were set at 0.20 and 0.34. Therefore, the minimum and maximum estimates for annual abortion rate were about 10.75 and 6.33 among female aged 15 to 44 years old, respectively.

Our current results indicated that both of those parameters (i.e. C and VF) were invalid. However, as these two parameters work in the opposite directions, they partially neutralized the effect of each other. Our current estimate, when data based and game of contact methods were used to estimate C and VF, is close to our maximum national estimate. It was the case when either aggregate sample or gender specific samples were analysed.

There are other indirect methods which can be applied to estimate size of stigmatized behaviors. This includes Cross-Wise, Proxy Respondents, and Item List. We should emphasize that the aim of this manuscript is neither to provide the abortion statistics in Iran nor to compare performance of other indirect size estimation methods with NSU.

One of the limitations of our study was that we only adjusted the estimates to address the visibility. There are other sources of error such as barrier effects [12]. One of assumptions of NSU is that members of hidden group can penetrate into our networks, and their network size is more or less is similar to that of the general population. This means that we have equal chance to know members of hidden group (barrier effect). To take into account the barrier effect, relative network size of hidden group to that of the general population should be calculated. This statistic, named Popularity Ratio (PR), combines information from the sample of the target group with that of the general population. PR has been estimated for FSW and PWID groups [12]. In this study, we assumed that there is no rational to assume that those experienced abortion are a marginalized population with smaller network size. Therefore, PF correction factor was not calculated.

Another limitation of our study was the method of sampling. While in Western cultures household or telephone based surveys are practical, our previous experience revealed that in the case of sensitive issues such methods lead to under-reporting [27]. Household surveys are popular as it is possible to get a representative sample. On the other hand, in Iranian culture, accuracy of replies in this method is low. Therefore, as a trade-off between representativeness and accuracy of replies, we applied a street based sampling scheme in which pedestrians were approached.

One of strengths of our study was that we applied VF which was derived from a sample of female who had abortion. On the other hand, in the national study experts’ estimate was applied. Those experts were selected from across the country. Although, experts’ opinion should guide the study, we have seen that this group might overestimate the visibility, as they are in touch with the abortion candidates.

Another important strength of our study is that we calculated age-gender distribution of network size and used the exact network size in the calculations. To our knowledge, no similar study has provided age-gender distribution of network size.

## Conclusion

To our knowledge, this was the first study to perform a multi-way sensitivity analysis to address the influence of the method of the C estimation, the VF and the study population on the size estimation of the age-gender specific hidden characteristics. We have seen that the determination of the age-sex distribution of the network size should be a prime. In addition, the selected sample does not affect the size if the sample-specific parameters are substituted in the formulas.

## Supporting information

S1 Data The dataset of the manuscript. (SAV)
